# Supplementary material for: Network inference from perturbation time course data
Source: NPJ Syst Biol Appl. 2022 Nov 1;8:42. doi: 10.1038/s41540-022-00253-6 (PMC9622863; doi:10.1038/s41540-022-00253-6)
Supplement: Supplementary file 1 — Supplementary Figures [file 41540_2022_253_MOESM1_ESM.pdf]

Sarmah et al., 2022. Supplementary Figure 1

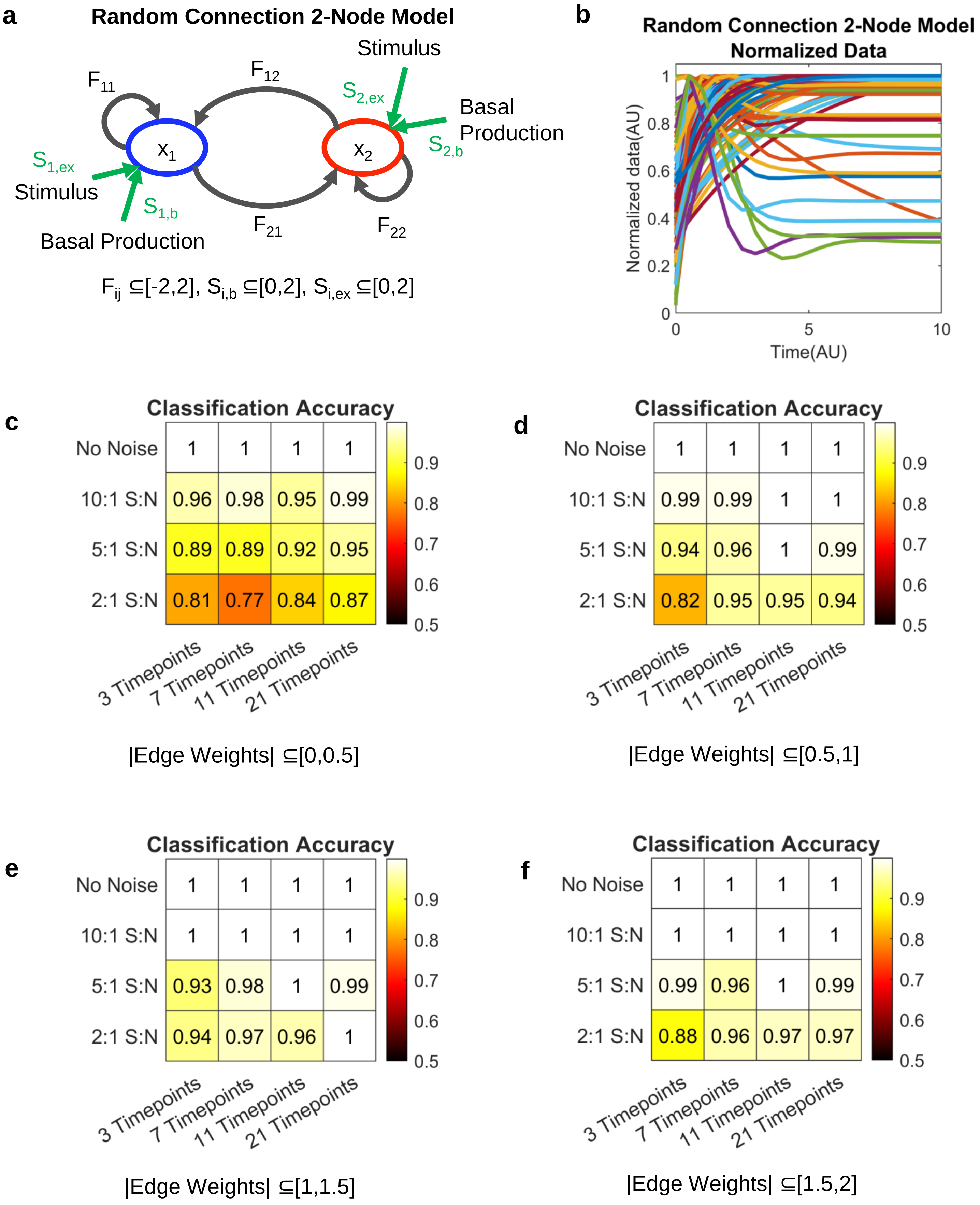

Supplementary Figure 1: (a) Random 2 node network with Jacobian elements labeled. Green arrows are basal production and external stimulus terms. **(b)** Time courses for the 50 random 2 node networks, normalized by the maximum value **(c,d,e,f)** Fraction of network parameters correctly classified in 50 randomly generated 2 node networks with an absolute value between 0 to 0.5 (c), 0.5 to 1 (d), 1 to 1.5 (e) and 1.5 to 2 (f).

Sarmah et al., 2022. Supplementary Figure 2

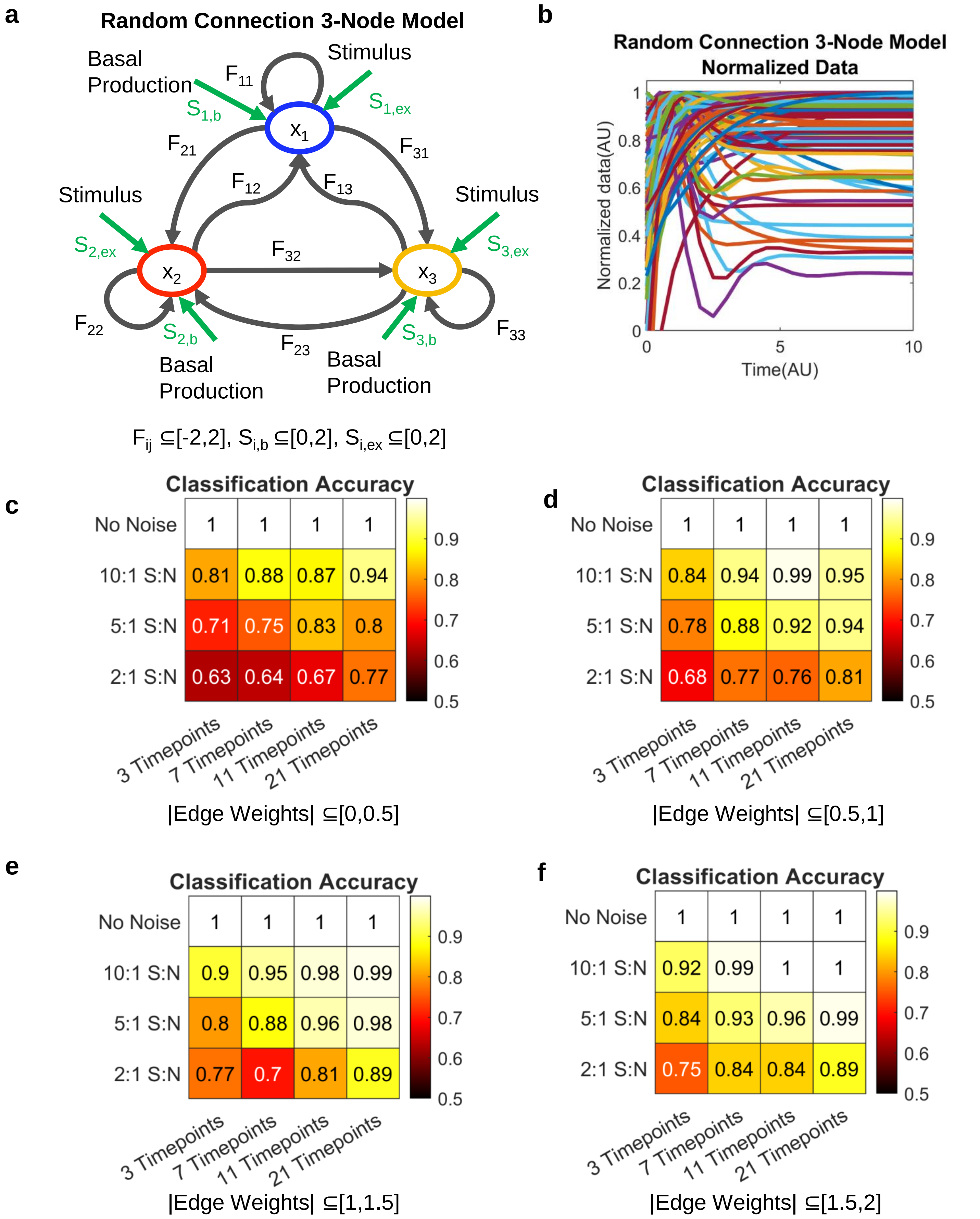

Supplementary Figure 2: (a) Random 3 node network with Jacobian elements labeled. Green arrows are basal production and external stimulus terms. **(b)** Time courses for 50 random 3 node networks, normalized by the maximum value **(c,d,e,f)** Fraction of network parameters correctly classified in 50 randomly generated 3 node networks with an absolute value between 0 to 0.5 (c), 0.5 to 1 (d), 1 to 1.5 (e) and 1.5 to 2 (f).

a

Random Connection 2-Node Models With Oscillatory behaviour

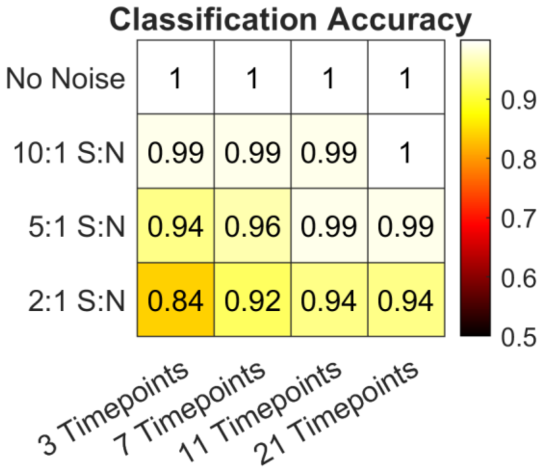

b

Random Connection 3-Node Models With Oscillatory behaviour

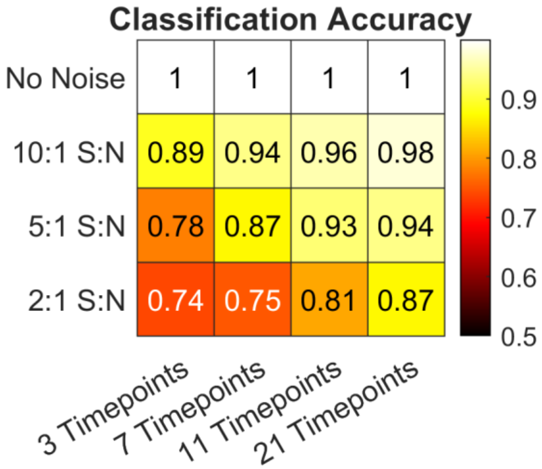

Supplementary Figure 3: (a,b) Fraction of network parameters correctly classified in 29 two node networks (a) and 29 three node networks (b) with potential for oscillatory behavior (non-zero imaginary parts of eigenvalues of Jacobian elements).

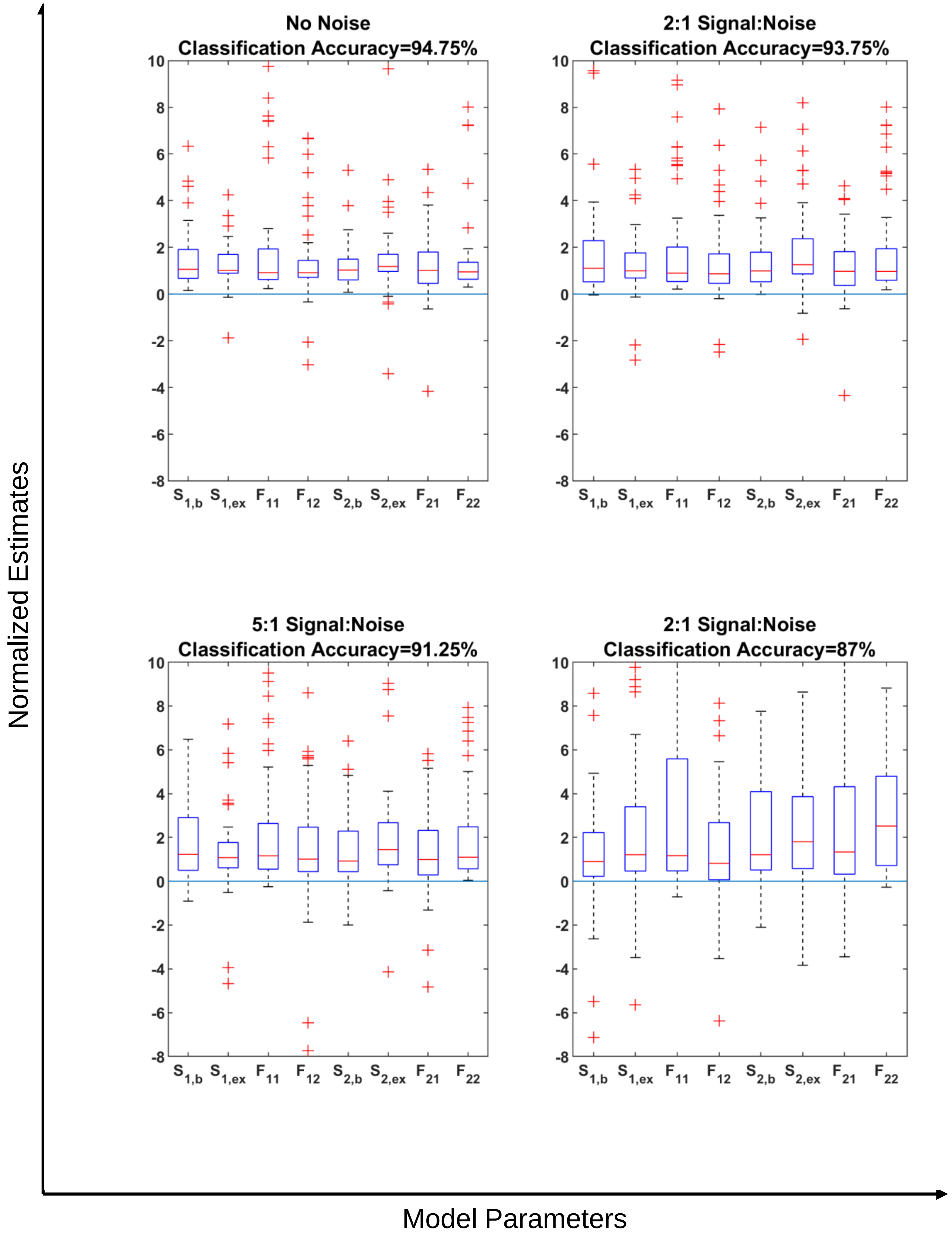

Supplementary Figure 4: Distribution of estimated parameters in two node networks normalized to corresponding actual parameters in 50 random three node systems, when the data from only node 1 and node 2 is included to make the estimation. A value of 1 means the parameters estimate did not change.

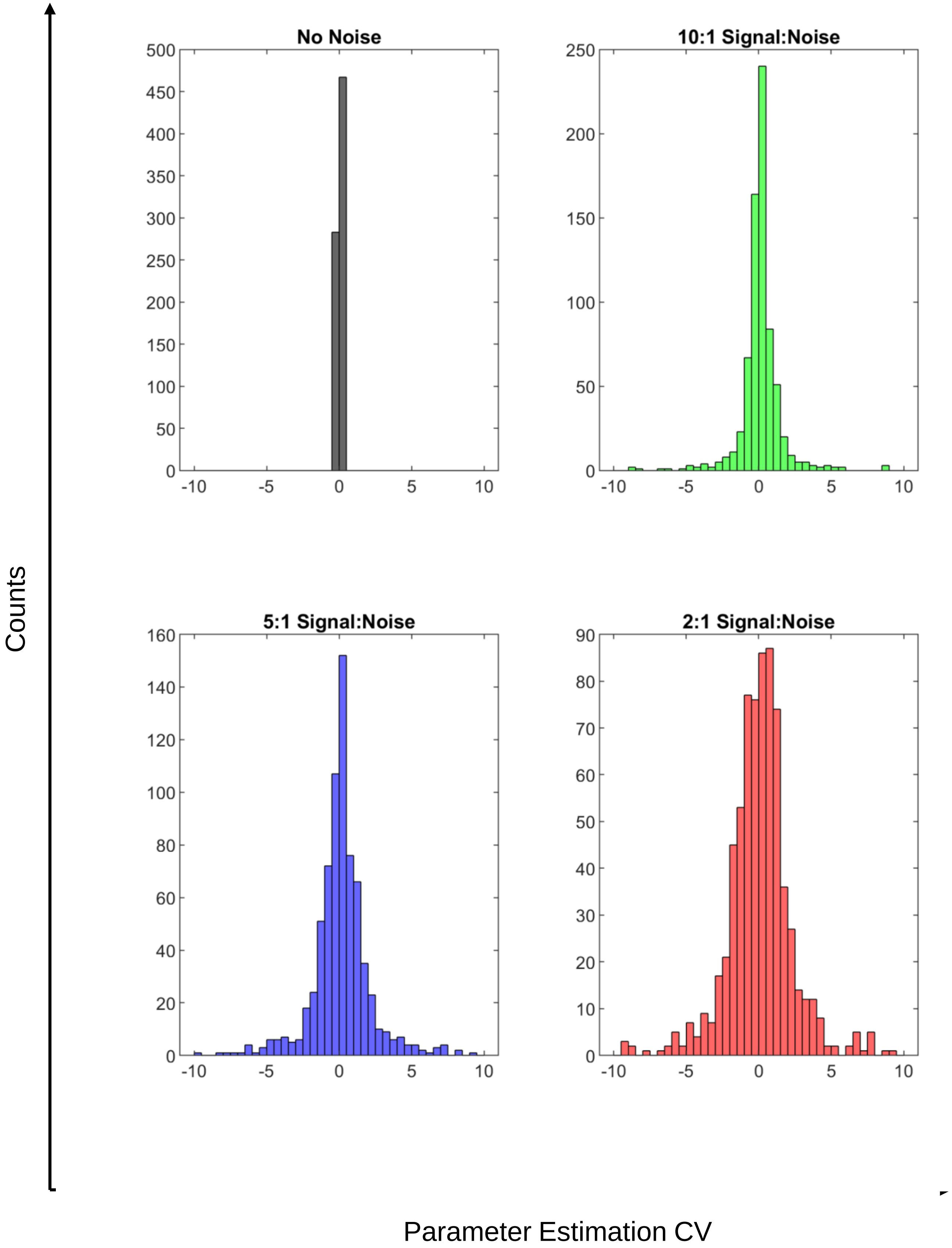

Supplementary Figure 5: Histogram of coefficient of variation (CV) among the parameters from multi start results in the 50 random three node models. Only parameter sets with sum of squared errors (SSE) less than twice the minimum SSE were included as acceptable.

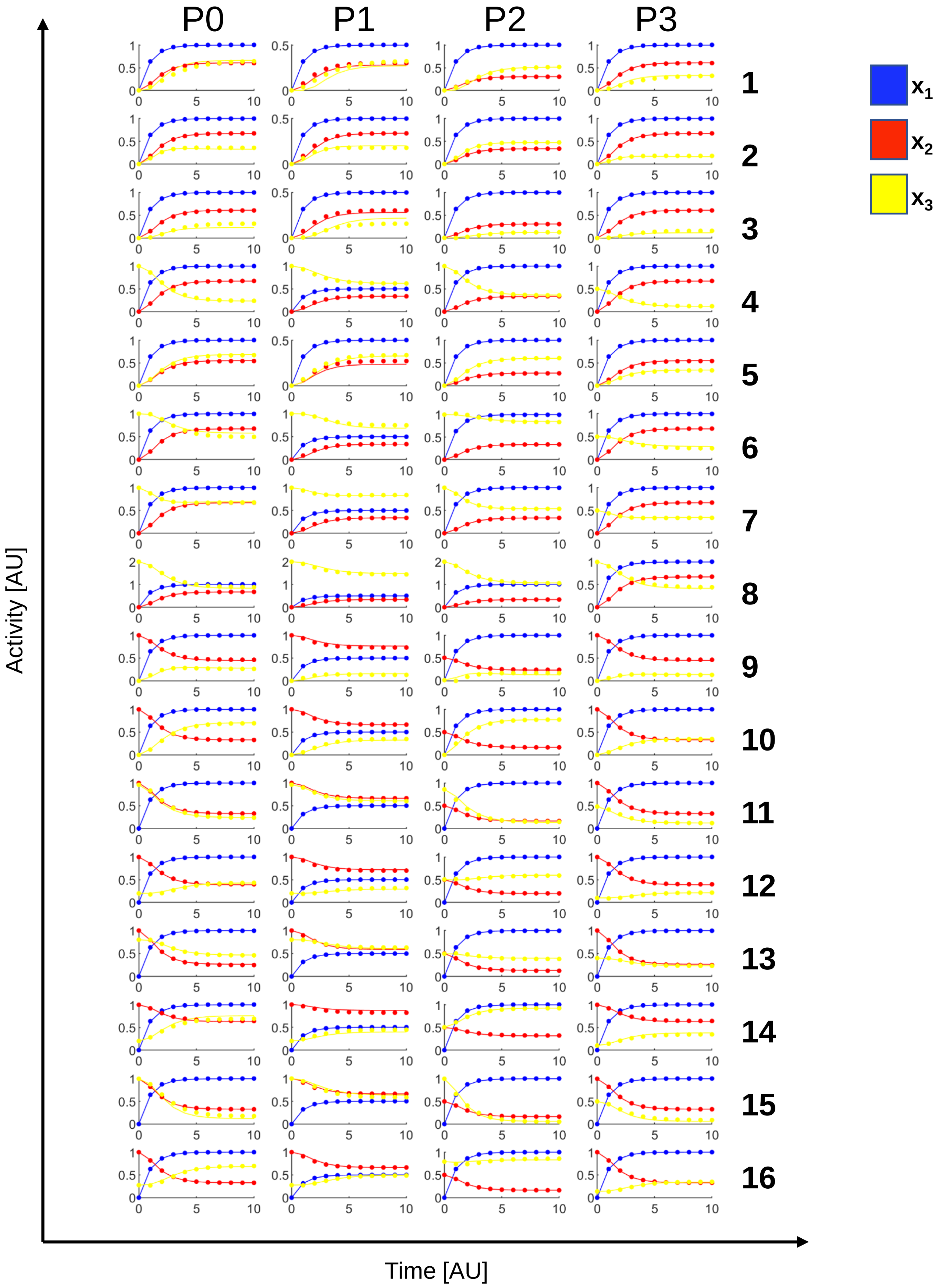

Supplementary Figure 6: Simulated, noise-free experimental data (dots) and model-generated fits (lines) for each FFL model structure. Different perturbations—vehicle (P0), perturb x1 (P1), perturb x2 (P2), perturb x3 (P3)—are across the columns and different model structures (1-16) are down the rows. Each node (1-3) is a different color as indicated in the legend.

Sarmah et al., 2022. Supplementary Figure 7

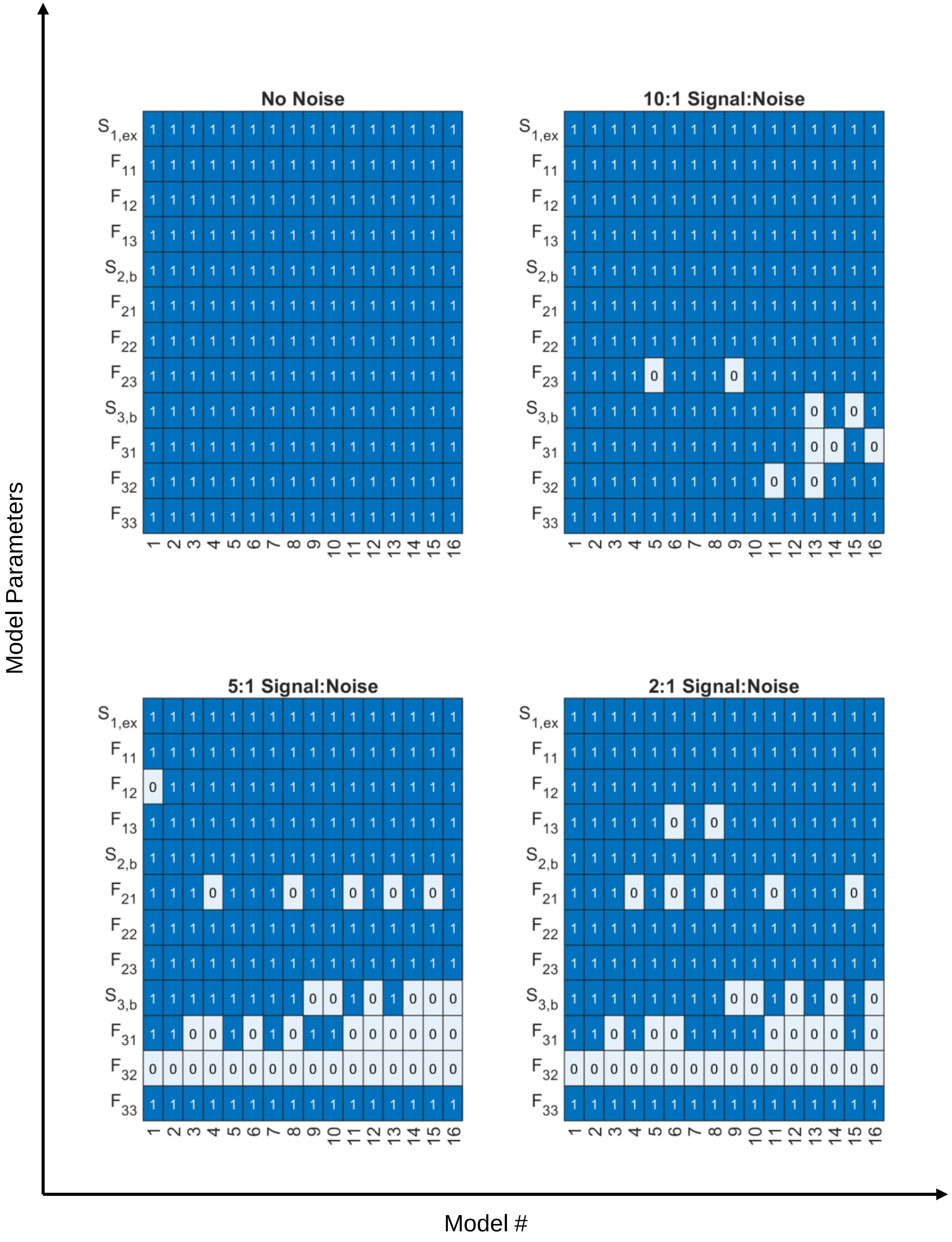

Supplementary Figure 7: Detailed results from the FFL models depicting whether a model parameter was correctly (1) or incorrectly (0) predicted for each model structure (1-16) under each noise level. Correct predictions were classified based on the optimal percentile cutoffs identified for each noise level.
